# Sex-specific machine learning classification models improve outcome prediction for abdominal aortic aneurysms

**DOI:** 10.1186/s13293-025-00765-w

**Published:** 2025-11-11

**Authors:** Katherine E. Kerr, Indrani Sen, Pete H. Gueldner, Tiziano Tallarita, Joseph C. Wildenberg, Nathan L. Liang, David A. Vorp, Timothy K. Chung

**Affiliations:** 1 Department of Bioengineering, University of Pittsburgh, Pittsburgh, PA USA; 2 Division of Cardiovascular Surgery, Mayo Clinic Health Systems, Eau Claire, Wisconsin, USA; 3 Division of Interventional Radiology, Mayo Clinic Health Systems, Eau Claire, Wisconsin, USA; 4 Department of Surgery, University of Pittsburgh, Pittsburgh PA, USA; 5 Division of Vascular Surgery, University of Pittsburgh, Pittsburgh PA, USA; 6 School of Medicine, University of Pittsburgh Medical Center, Pittsburgh, PA, USA; 7 McGowan Institute for Regenerative Medicine, University of Pittsburgh, Pittsburgh,, PA, USA; 8 Department of Chemical and Petroleum Engineering, University of Pittsburgh, PA, USA; 9 Department of Cardiothoracic Surgery, University of Pittsburgh, Pittsburgh, PA, USA; 10 Clinical & Translational Sciences Institute, University of Pittsburgh, Pittsburgh, PA, USA; 11 Department of Mechanical Engineering and Materials Science, University of Pittsburgh, Pittsburgh, PA, USA; 12 Center for Vascular Remodeling and Regeneration, University of Pittsburgh, Pittsburgh, PA, USA; 13 Magee Womens Research Institute, University of Pittsburgh, Pittsburgh, PA, USA; 14 Department of Mechanical Engineering, University of Texas San Antonio, Texas San Antonio, United States of America

**Keywords:** Abdominal aortic aneurysm, Machine learning, Sex-based differences, Outcome prediction, AI explainability, Biomechanics, Morphology, Stress analysis, Shape analysis, Vascular surgery

## Abstract

**Background:**

Abdominal aortic aneurysm (AAA) is an abnormal dilation of the abdominal aorta that carries up to a 90% mortality rate when ruptured. Although male patients experience AAA at a higher rate than females, female patients experience AAA rupture at a rate three- to four-fold higher that of their male counterparts. The current standard clinicians use for determining when to surgically intervene is maximum transverse diameter of the AAA perpendicular to the axis of flow. However, some aneurysms below these diameter thresholds rupture. Machine learning (ML) classification models have been previously shown to predict patient outcomes with more discriminability than the diameter criterion. However, these models do not consider sex-based differences. In this proof-of-concept study, we investigate how creating sex-specific ML models impacts patient outcome prediction as compared to a general model encompassing all patients (sex agnostic).

**Methods:**

Computed tomography image sets were acquired from 537 patients (*n* = 159 female, *n* = 378 male) at the University of Pittsburgh Medical Center (UPMC) and Mayo Clinic Health Systems. Features used as input to the ML models were categorized as clinical, biomechanical, and morphological data. Prior to ML model training, patient data were randomly split for 20% holdout testing. ML models encompassing all patients (general model), only male patients (male-specific model), and only female patients (female-specific model) were trained and tested.

**Results:**

A female-specific model and male-specific model both had a higher maximum area under the receiver-operating characteristic curve values than a general model for female patients and male patients, respectively. Equalizing the sample sizes of female and male patients in the model led to improved outcomes for female patients without decreasing performance for male patients.

**Conclusion:**

ML classification models show promise in improving predictions of patient outcomes for AAA. The higher AAA prevalence rate for males leads to female patients being underrepresented in AAA datasets. In this proof-of-concept study, we demonstrated that sex-specific models outperformed a general model in predicting patient outcomes. Additionally, equalizing sample sizes within the dataset improved predictions for female patients without compromising overall performance of the model. As ML applications in medicine continue to grow, it is important to consider population representation within datasets to reduce model bias.

**Supplementary Information:**

The online version contains supplementary material available at 10.1186/s13293-025-00765-w.

## Highlights

- Although abdominal aortic aneurysm has a higher prevalence rate in male patients, female patients experience higher rupture rates as well as higher surgical and post-operative mortality rates, indicating a need for sex-based considerations in outcome prediction.
- This work demonstrates improved outcome predictions when patient models are striated based on sex for female and male patients as well as sex-specific differences in feature importance, suggesting that grouping by sex should be considered in ML models for AAA outcome prediction.
- As machine learning tools continue to grow in prevalence within the medical environment, accounting for bias within these models is crucial to ensure generalizability.

**Supplementary Information:**

The online version contains supplementary material available at 10.1186/s13293-025-00765-w.

## Abstract (plain language summary)

Ballooning of the aorta, a major artery that delivers blood from the heart to the body, in the abdomen, or an abdominal aortic aneurysm, is often asymptomatic, but can be deadly if ruptured. However, not all abdominal aortic aneurysms rupture. Predicting if rupture will occur is important, as surgery can prevent rupture, but also comes with its own risks. Clinicians currently use the diameter of the aneurysm to decide when to perform surgery, but diameter does not always accurately predict ruptures. Men get abdominal aortic aneurysms more than women do, but women have a higher rate of these ruptures. There have been some machine learning tools that have been designed to predict when these ruptures occur, but they do not account for differences between men and women in their models. In this work, tools were created that did a better job of predicting whether or not an aneurysm will rupture than diameter. The tools that were sex-specific did a better job than the ones that encompassed all patients.

**Supplementary Information:**

The online version contains supplementary material available at 10.1186/s13293-025-00765-w.

## Introduction

Abdominal aortic aneurysm (AAA) is an abnormal focal dilation of the infrarenal aorta to at least 50% of its normal size. Rupture of AAA can be devastating as it carries a mortality rate of up to 90% [1, 2]. Although men experience a higher prevalence of AAA [3], women experience higher AAA rupture rates, with literature reporting a rupture rate 3 to 4 times higher for females than that of males [1, 4–6]. It has also been reported that female patients experience higher growth rates [7] as well as experience AAA rupture at smaller diameters [8]. Additionally, female patients who have undergone surgical repair for their AAA experience longer intensive care unit stays after intervention, higher surgical and postoperative mortality, and higher 5-year endovascular repair mortality and reintervention rates [9–11].

Due to its associated high mortality rate, it is important to accurately determine which patients are at risk of AAA rupture. Currently, clinicians primarily rely on the maximum transverse diameter of the AAA, with the current recommendations for surgical intervention being 5.0 cm for female patients and 5.5 cm for male patients [12]. However, studies have previously reported that between 7 and 23.4% of aneurysms between 4.1 and 5.5 cm rupture [13–15]. Additionally, it has been previously shown that diameter predicts AAA rupture with an accuracy of 73% [16].

The shortcomings of AAA maximum transverse diameter as a diagnostic criterion have led to investigation into multiple biomechanical and morphological indices to predict AAA outcome. Peak wall stress was previously proposed as a predictor of AAA outcome (stable vs. surgical intervention) [16], however heterogeneity in wall strength both within a given aneurysm and between patients make using peak wall stress alone to predict outcome unreliable [14]. Morphological indices such as the aortic size index (the ratio of patient AAA diameter to patient body surface area) [17] have shown improved outcome prediction potential for female patients [18], but is not a better predictor than diameter in all cases [19]. The ratio of locally acting wall stress to strength, or rupture potential index (RPI) [20], showed promise in predicting AAA outcome [16, 20–22] but clinical adoption was limited due to the time and expertise required to perform reconstructions and finite element stress analyses. Using automated image-based analyses to acquire biomechanical and morphological parameters no longer requires time intensive segmentation, however, making clinical adoption more feasible.

Machine learning (ML) classification models have shown promise in predicting AAA outcomes [23, 24]. Previous work from our group has demonstrated that ML models incorporating clinical, morphological, and biomechanical factors outperform diameter alone in discriminability of AAA outcomes [24]. However, these models did not account for any sex-based factors in their predictions, such as the natural overrepresentation in the number of male patients in AAA datasets due to higher AAA prevalence rates among male patients compared to female patients.

In this work, we create sex-based ML models and compare their ability to predict outcomes to a general model agnostic of sex. This includes training models exclusively using data from female or male patients as well as equalizing the sample sizes of male and female patients. We then explore how incorporation of these sex-based differences impacts patient-specific prediction of AAA outcomes.

## Methods

### Data acquisition

Data were acquired from patients seen at the University of Pittsburgh Medical Center (UPMC) and the Mayo Clinic Health System. AAA patients were identified using international classification of disease (ICD) 9/10 and current procedural terminology (CPT) codes with chart review to confirm diagnosis. Patients from UPMC were obtained from a retrospective database from the Health Record Research Request, a service of the University of Pittsburgh Department of Bioinformatics in partnership with UPMC, for cases between 2004 and 2019. Deidentified patient computed tomography (CT) images within the Mayo Health system between 2021 and 2023 were extracted and delivered through a data use agreement #DUA00004445 to the University of Pittsburgh for analysis. This proof-of-concept study consisted of a total of 537 patients, of which 159 (29.6%) were female and 378 (70.3%) were male. Patients had CT scans over a duration of 5.69 ± 3.37 years. AAAs that were in the “repair” or “rupture” groups either were deemed at risk of rupture and underwent interventional repair or experienced rupture, respectively, within their follow-up duration. All others were put in the “stable” group, meaning that they did not result in repair or rupture within their follow up timeframe. The male cohort included 313 stable patients, 55 repair, and 10 rupture. The female patient groups consisted of 123 stable patients, 31 repair, and 5 rupture. To mitigate the class imbalance between the repair and the rupture group compared to the stable group, we pooled the repair and rupture groups together into one “unstable” group per sex (65 male, 36 female).

### Image segmentation, reconstruction, and morphological analysis

Our image segmentation pipeline has been previously published [24–26]. Briefly, Digital Imaging and Communications in Medicine (DICOM) image stacks were automatically segmented using a U-NET image classifier. This U-NET image classifier was previously trained by our group using Amazon Web Service’s (Amazon, Seattle, WA, USA) Elastic Compute Cloud [25]. Semi-automatic methods were utilized for image sets that failed to be segmented using automatic methods. A binary mask was created for the wall, lumen, and intraluminal thrombus (ILT) regions from the segmented image. A point cloud was then created of each axial slice and the original spacing of the CT image was registered and scaled. The point cloud was then converted into a mesh using Poisson 3D surface reconstruction [24, 27]. From there, post-processing including smoothing of the meshes was performed before morphological analysis was completed [24, 26].

Morphological indices were extracted using a previously described custom in-house MATLAB (MathWorks Inc., Natick, MA, USA) script (one-, two-, three-, and higher dimensional indices) [24, 26]. A table of morphological indices that were extracted has been previously published [24].

### Biomechanical analysis

The stress analysis performed used a well-established computational pipeline to automatically assign material properties to the AAA wall and any intraluminal thrombus (ILT) that was present [24, 26]. Briefly, the wall stress analysis was performed by constraining the proximal and distal boundaries of the AAA and pressurizing to an ideal systolic pressure of 120 mmHg. A uniform wall thickness of 1.9 mm was assumed [24]. Models previously validated for ILT (hyperelastic and isotropic) [28, 29] and AAA wall (hyperelastic and anisotropic) [30] were utilized. After performing the simulation in Abaqus Standard (implicit mode) (ABAQUS Inc., Providence, Rhode Island, United States), the mean and peak von Mises wall stresses were calculated from the isolated aneurysm sac regions [26].

### Statistical tests

Clinical, biomechanical, and morphological data were checked for normality using the Kolmogorov–Smirnov test. The only normal dataset was the age at outcome, so age was therefore compared using a two-tailed t-test. As all other data were not normal, statistical comparisons of various clinical, biomechanical, and morphological indices between female and male cohorts were performed using a two-tailed Mann-Whitney non-parametric test. Clinical variables with binary outcomes were compared between female and male groups using a Fisher’s exact test.

### Machine learning models

The dataset was split 80%−20% randomly a priori, controlling for stable and unstable cases, to use 20% for holdout testing. The dataset was split 5 separate times to control whether data splitting had any impact on model performance (i.e., to investigate the dataset stability). A ML classification model was trained in MATLAB as well as in Python using XGBoost. Based on the imbalance between the number of stable and unstable cases in the cohort, an ensemble boosted model (Random Under Sampling (RUS) Boost) was used. A “general” model was trained using all patients in the cohort (both sexes) while the sex-specific models were trained using “female” and “male” data separately. The performance of female patients and male patients in the general model was found by extracting the results of female and male patients separately from the general model. Receiver operating characteristic (ROC) curves and confusion matrices were used to visualize the model’s ability to predict unstable vs. stable AAA cases. Feature importance between the models was compared using Gini feature importance within XGBoost. Feature importance values are normalized to all values within a specific model. Each value represents the amount of node impurity that is reduced within a tree based on a given parameter. For further local and global explainability, Shapley Additive Explanations (SHAP) values were created for each model [31]. SHAP values are calculated for all clinical, biomechanical, and morphological indices based on the relative impacton individual predictions compared to all other features [32]. Additionally, a model was trained and tested with an equal number of female and male patients that were matched based on diameter and compared to the general model results.

## Results

### Comparisons of clinical, morphological, and biomechanical parameters

There were no significant differences in clinical indices between male and female patients (Table 1A). The race of two male patients was missing from the dataset and is therefore not included in the table. However, we did see significant differences in some morphological parameter values, including lumen and wall surface area, maximum diameter, and both the mean and maximum values for both lumen and wall asymmetry being lower in females vs. males (Table 1B). Female patients also had lower mean and peak wall stress, as well as lower mean and peak tension, compared with male patients (see Table 1C).

### Model training results for general and sex-specific models

**Fig. 1:**
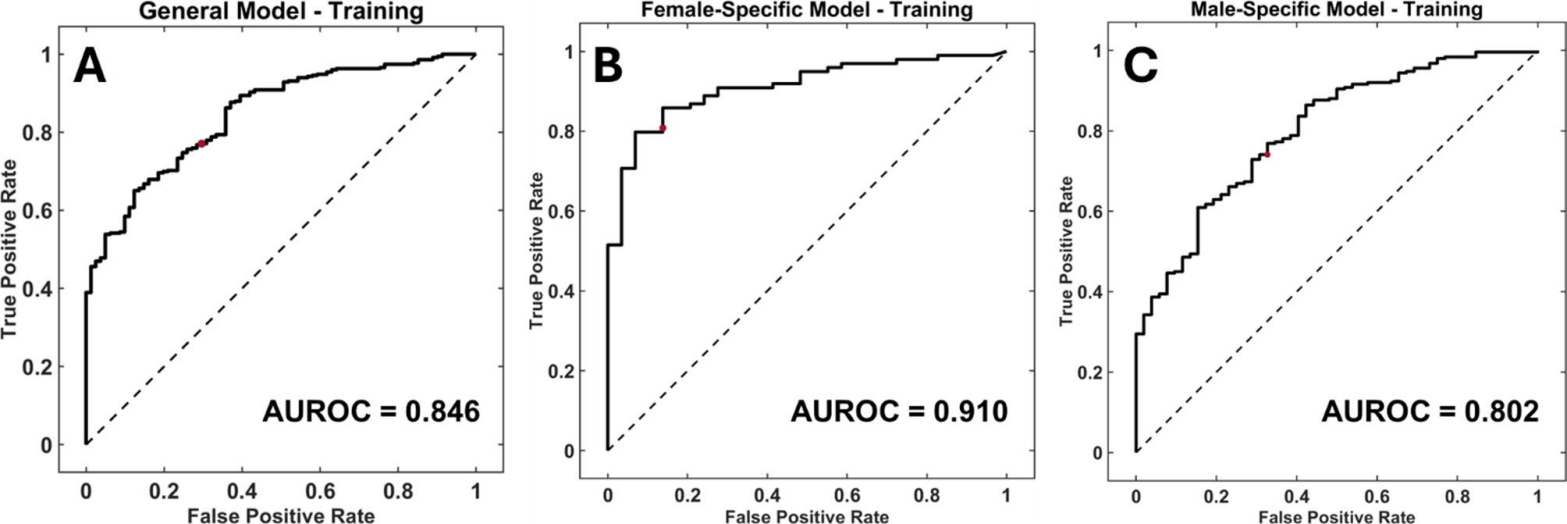
Model Training Receiver-Operating Characteristic Curves. Receiver-operating characteristic (ROC) curves for the model training dataset. **(A)** General model (a sex-agnostic model including all patients) training ROC with an area under the ROC curve (AUROC) value of 0.846. **(B)** Female-specific model training ROC with an AUROC value of 0.910. **(C)** Male-specific model training ROC with an AUROC value of 0.802

Models were trained 5 separate times on 5 different splits of the dataset to ensure the split of the data was not skewing results. The general, female-specific, and male-specific accuracies and area under the ROC curve (AUROC) (Fig. 1) can be seen in Table 2. All accuracies and AUROC values for the training set were above 66.3% and 0.715, respectively. We chose to proceed with testing our models based on these results.

**Table 1:** Clinical, Morphological, and Biomechanical data

		Female (*n* = 159)	Male (*n* = 378)	*P* value
**A. Clinical Data**				
Age at Diagnosis (years)		69.43 ± 10.49	69.85 ± 8.73	0.97
Age at Event (years)		74.70 ± 9.70	75.58 ± 8.92	0.31
Time to Outcome or Follow Up (years)		5.58 ± 3.40	5.73 ± 3.29	0.64
Chronic Kidney Disease		37.11% (*n* = 59)	35.19% (*n* = 133)	0.69
Coronary Artery Disease		62.26% (*n* = 99)	69.84% (*n* = 264)	0.11
Diabetes		35.22% (*n* = 56)	39.95% (*n* = 151)	0.33
Hyperlipidemia		78.62% (*n* = 125)	80.16% (*n* = 303)	0.72
Hypertension		90.57% (*n* = 144)	90.48% (*n* = 342)	1.00
Peripheral Artery Disease		41.51% (*n* = 66)	46.56% (*n* = 176)	0.30
Race (*n* = 159 female, *n* = 376 male, 2 males with race unavailable)	White (*n* = 491)	89.31% (*n* = 142)	92.82% (*n* = 349)	0.22
	Black (*n* = 42)	10.06% (*n* = 16)	6.91% (*n* = 26)	
	Asian (*n* = 1)	0.00% (*n* = 0)	0.27% (*n* = 1)	
	Hispanic (*n* = 1)	0.63% (*n* = 1)	0.00% (*n* = 0)	
Aspirin		62.89% (*n* = 100)	61.38% (*n* = 232)	0.77
Plavix		36.00% (*n* = 54, 150 total)	38.36% (*n* = 145)	0.69
Statin		47.80% (*n* = 76)	55.56% (*n* = 210)	0.11
**B. Morphological Data**				
Max Diameter (cm)		4.36 ± 1.59	4.80 ± 1.25	< 0.001
Max Intraluminal Thrombus Thickness (mm)		14.68 ± 13.07	17.31 ± 13.00	0.029
Lumen Surface Area (cm^2^)		77.75 ± 30.82	92.06 ± 30.93	< 0.001
Wall Surface Area (cm^2^)		104.64 ± 50.53	120.42 ± 47.37	< 0.001
Max Lumen Asymmetry		47.00 ± 46.88	58.47 ± 53.05	0.019
Max Wall Asymmetry		44.88 ± 46.96	58.73 ± 52.58	0.003
Mean Lumen Asymmetry		24.64 ± 23.99	30.14 ± 26.73	0.046
Mean Wall Asymmetry		23.85 ± 24.00	30.37 ± 26.42	0.009
Lumen Volume (ml)		36.87 ± 24.97	47.31 ± 28.61	< 0.001
Wall Volume (ml)		66.41 ± 56.53	78.70 ± 58.12	< 0.001
Neck Height (cm)		5.66 ± 3.00	6.27 ± 3.29	0.044
Sac Height (cm)		18.95 ± 5.02	20.99 ± 5.07	< 0.001
**C. Biomechanical Data**				
Mean Wall Stress (N/cm^2^)		8.71 ± 2.95	10.00 ± 2.83	< 0.001
Peak Wall Stress (N/cm^2^)		17.47 ± 6.33	20.65 ± 6.43	< 0.001
Peak Wall Tension (N/cm)		26.54 ± 12.00	30.56 ± 11.70	< 0.001
Mean Wall Tension (N/cm)		12.93 ± 4.87	14.62 ± 4.83	< 0.001

Comparisons of selected model metrics between female and male patients **(A)** Clinical data for the cohort striated by female and male patients. The race of two male patients was not included in the clinical data and is not represented in this table. **(B)** Morphological data for the cohort striated by female and male patients. **(C)** Mean and peak wall tensions and wall stresses for female and male patients

**Table 2:** Model training results

General		Female-Specific		Male-Specific	
AUROC	Accuracy	AUROC	Accuracy	AUROC	Accuracy
0.810	74.2%	0.847	81.2%	0.760	67.7%
0.846	75.8%	0.910	82.0%	0.715	66.3%
0.824	75.3%	0.887	80.5%	0.782	71.9%
0.794	72.8%	0.868	79.7%	0.765	69.0%
0.798	72.8%	0.831	78.9%	0.802	72.9%

Model training results for the general (a sex-agnostic model including all patients), female-specific, and male-specific model. Area under the ROC curve (AUROC) and accuracy are provided for five different splits of the dataset

### Model testing results for general and sex-specific models

**Fig. 2:**
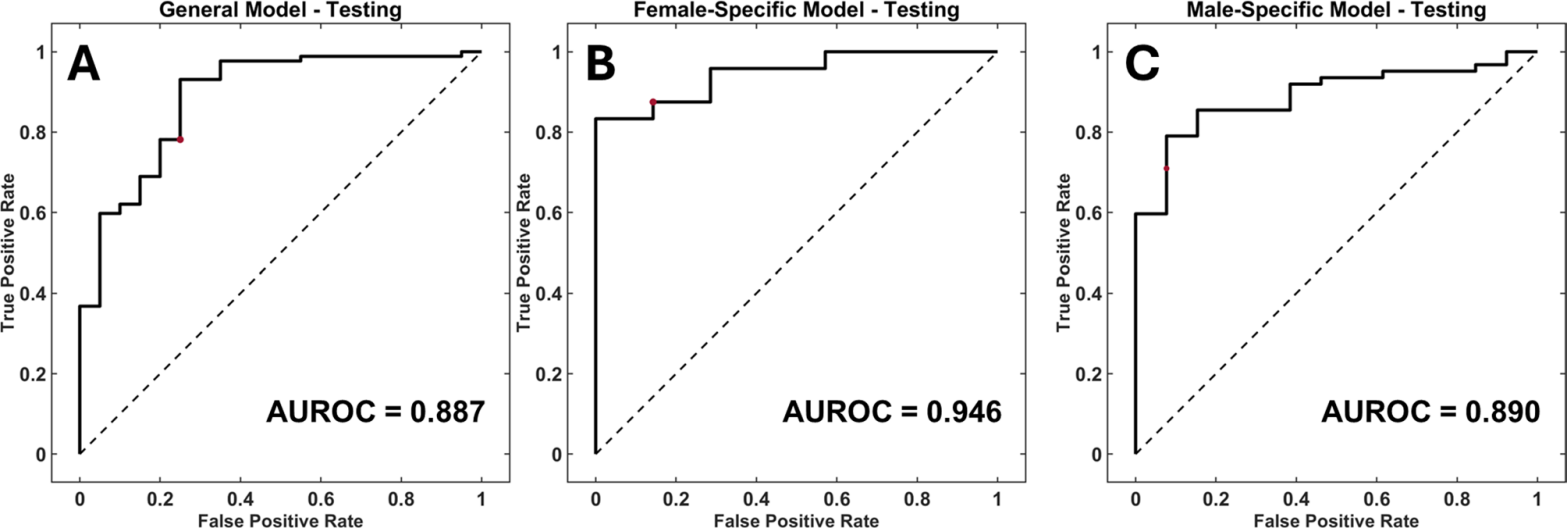
Model Testing Receiver-Operating Characteristic Curves. Receiver-operating characteristic (ROC) curves for the model testing dataset. **(A)** General model training ROC with an area under the ROC curve (AUROC) value of 0.887. **(B)** Female-specific model training ROC with an AUROC value of 0.946. **(C)** Male-specific model training ROC with an AUROC value of 0.890

Each model was tested using a holdout dataset consisting of 20% of the cohort for each separate model. The AUROCs and accuracies are reported in Table 3; Fig. 2. The female-specific model outperformed the female patients in the general model with a maximum AUROC of 0.946 and an accompanying accuracy of 87.1% compared to a maximum AUROC of 0.838 and an accompanying accuracy of 86.2%. The male-specific model (maximum AUROC 0.890, accompanying accuracy 80.0%) also had a higher AUROC and accuracy than the male patients in the general model (maximum AUROC 0.775, accompanying accuracy 73.6%).

**Table 3:** Testing results for general and Sex-Specific models

General Model		Female-Specific Model		Male-Specific Model		General Model – Female Patients		General Model – Male Patients	
AUROC	Accuracy	AUROC	Accuracy	AUROC	Accuracy	AUROC	Accuracy	AUROC	Accuracy
0.887	77.6%	0.946	87.1%	0.890	74.7%	0.817	85.3%	0.695	65.8%
0.878	78.5%	0.887	87.1%	0.839	80.0%	0.838	86.2%	0.698	71.8%
0.871	72.0%	0.860	83.9%	0.823	74.7%	0.794	88.6%	0.775	73.6%
0.840	77.6%	0.827	77.4%	0.809	68.0%	0.800	81.1%	0.745	75.7%
0.816	75.7%	0.821	74.2%	0.750	66.7%	0.760	86.7%	0.687	74.0%

Model testing results for the general (a sex-agnostic model including all patients), female-specific, and male-specific model as well as the female and male patients within the general model. AUROC and accuracy are provided for five different splits of the dataset

### Gini feature importance

**Fig. 3:**
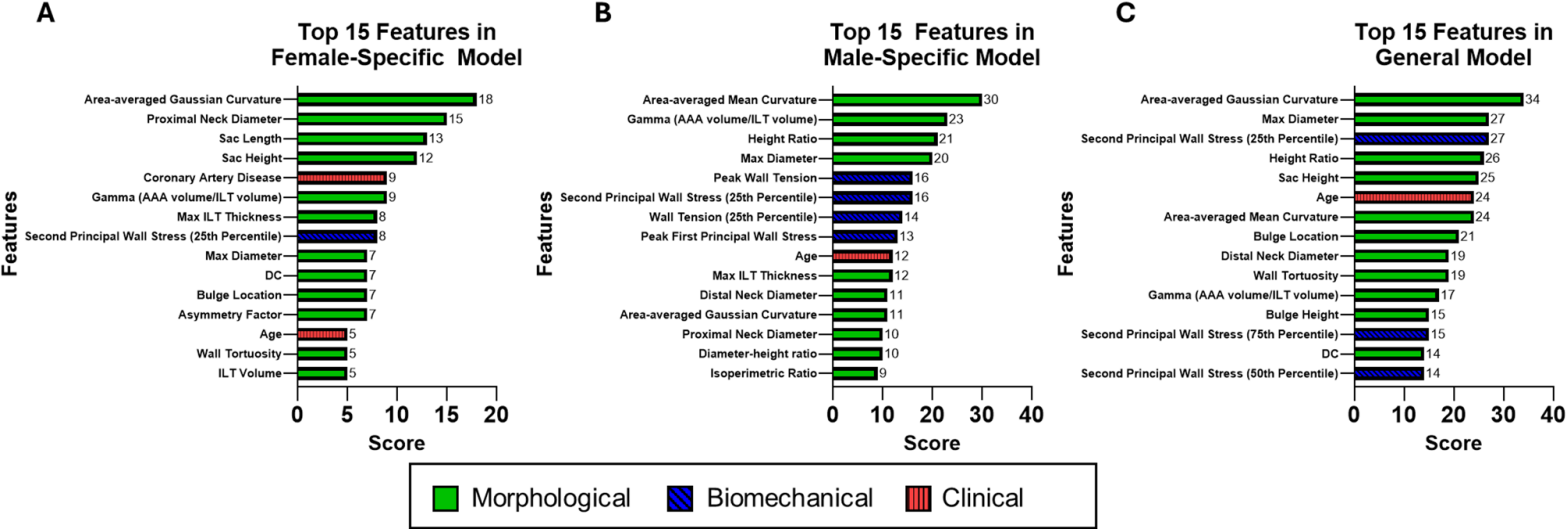
Feature Importance – Gini Feature Importance.The top 15 features for each model using Gini feature importance **(A)** Gini feature importance for the female-specific model. One out of the top 15 features was biomechanical (second principal wall stress (25th percentile)) and two were clinical (coronary artery disease and age). **(B)** Gini feature importance for the male-specific model. Four out of the top 15 features were biomechanical (peak wall tension, second principal wall stress (25th percentile), wall tension (25th percentile), peak first principal wall stress) and one was clinical (age). **(C)** Gini feature importance for the general model. Three out of the top 15 features were biomechanical (second principal wall stress (25th percentile), second principal wall stress (50th percentile), second principal wall stress (75th percentile)) and one was clinical (age)

**Fig. 4:**
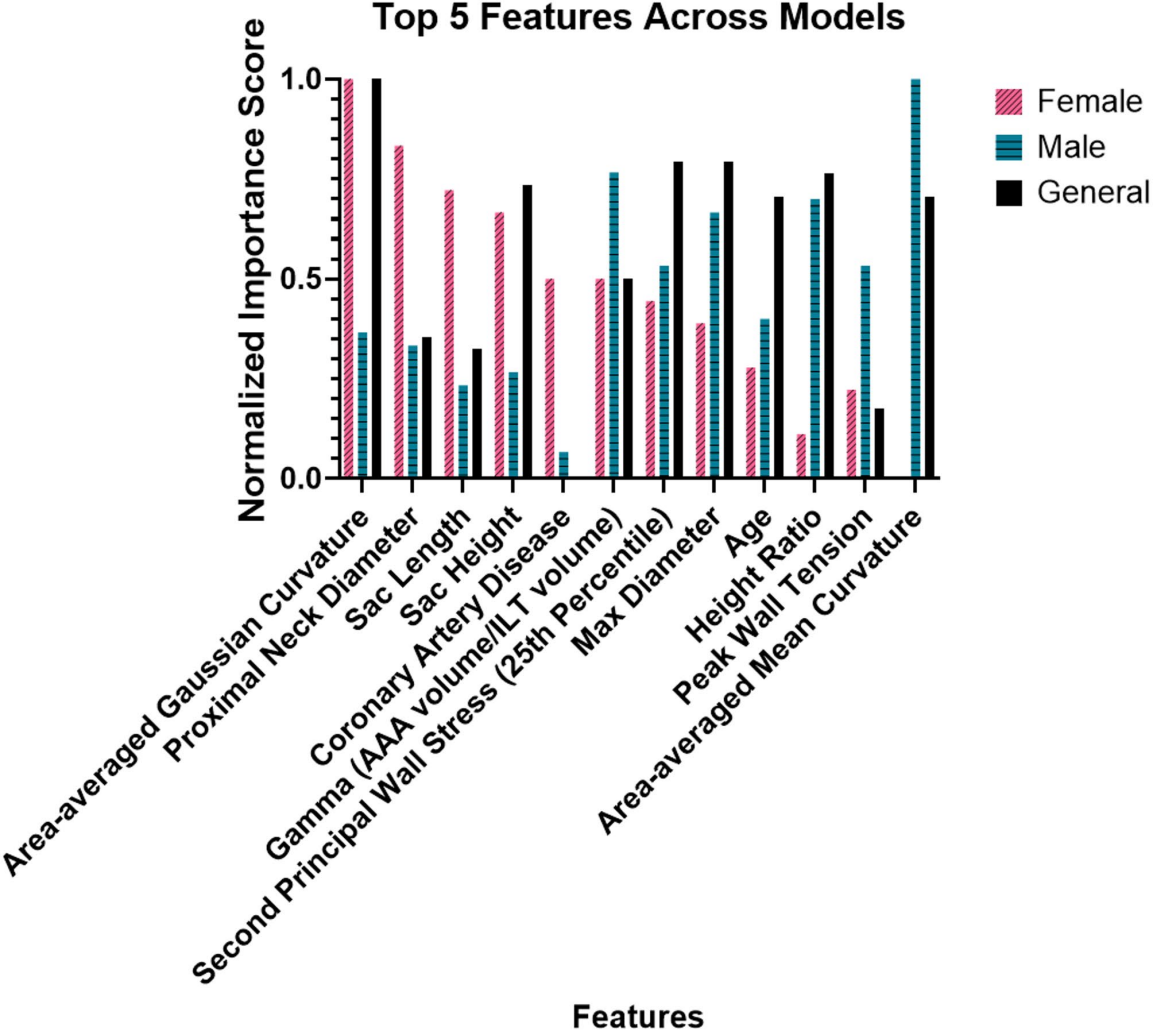
Comparing Feature Importance Across Models. A comparison of the top 5 features for the female-specific, male-specific, and general models. Feature importance values were normalized to the highest score for each model

Gini feature importance for the top 15 most important input parameters in terms of impact on the model’s prediction of stable vs. unstable AAA can be seen in Fig. 3A and C. For both the general and female-specific model, area-averaged Gaussian curvature was the top metric, while area-averaged mean curvature was the top metric for the male-specific model. Biomechanical predictors were more prevalent in the top 15 for both the male-specific and general model, with 4 and 3 out of the top 15 most important features being biomechanical predictors for the male and general model respectively, compared to only 1 biomechanical variable out of the top 15 for the female-specific model. The female-specific model included 2 clinical variables (age and coronary artery disease) compared to only 1 clinical variable (age) in the top 15 features in the male-specific and general model. A comparison of the top 5 features for each model can be found in Fig. 4.

### SHAP values

**Fig. 5:**
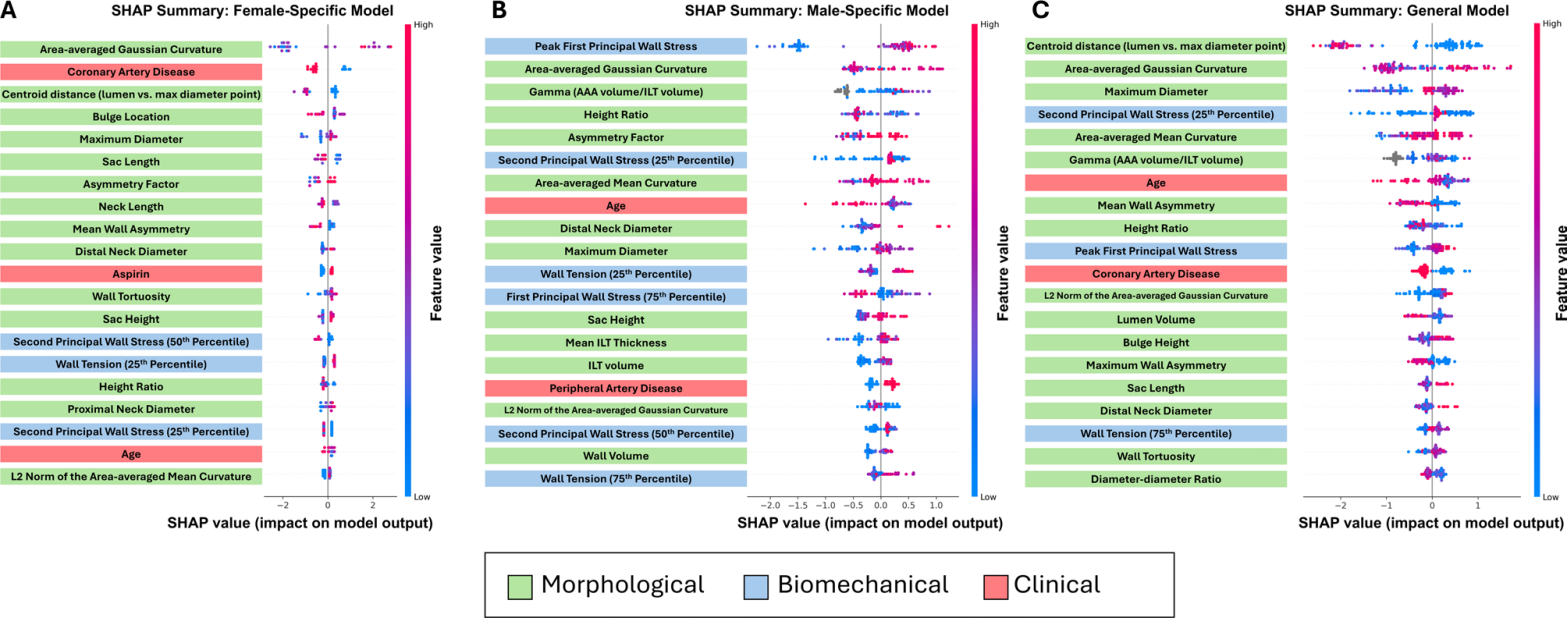
SHAP values for each model. The top 20 indices for each model using SHAP values (**A**) SHAP values for the female-specific model. Three out of the top 20 features were biomechanical (second principal wall stress (25th percentile), wall tension (25th percentile), and second principal wall stress (50th percentile)) and three were clinical (coronary artery disease, aspirin, and age). (**B**) SHAP values for the male-specific model. Six out of the top 20 features were biomechanical (peak first principal wall stress, second principal wall stress (25th percentile), wall tension (25th percentile), first principal wall stress (75th percentile), second principal wall stress (50th percentile), wall tension (25th percentile)) and two were clinical (age and peripheral artery disease). (**C**) SHAP values for the general model. Three out of the top 15 features were biomechanical (second principal wall stress (25th percentile), peak first principal wall stress, wall tension (75th percentile)) and two were clinical (age and coronary artery disease)

A beehive plot of the SHAP values for the top 20 features in each model can be seen in Fig. 5A and C, ranked top to bottom by impact on the model. Similar to the Gini feature importance, the female-specific model placed the most importance on morphological parameters, with the area-averaged Gaussian curvature being the top metric. The male model had a biomechanical parameter as its top metric, with peak first principal wall stress having the largest SHAP values. For the general model, the most importance was the centroid distance followed by the area-averaged Gaussian curvature. Biomechanical parameters played the largest role in the male-specific model, with six of the top 20 indices, compared to three for both the general and female-specific models.

### Equal sample sizes for female and male patients

Since one cause of decreased general model performance for female patients may be decreased representation due to higher prevalence of AAA in male patients, we created a model with equal sample sizes between male and female patients (Table 4). Male patients were diameter-matched to female patients to create the cohorts. The general model with equal sample sizes showed no notable differences from the general model including all patients. Female patients in the general model had lower AUROC and accuracies than the female patients in the general model with equal sample sizes. Male patients in the general model had similar accuracies and slightly lower AUROCs than the male patients in the general model with equal sample sizes.

**Table 4:** Testing results for the general model vs. the model with equal sample sizes

General Model		Equal Sample Sizes Model		General Model – Female Patients		Equal Sample Sizes - Female		General Model – Male Patients		Equal Sample Sizes - Male	
AUROC	Accuracy	AUROC	Accuracy	AUROC	Accuracy	AUROC	Accuracy	AUROC	Accuracy	AUROC	Accuracy
0.887	77.6%	0.900	85.70%	0.833	85.7%	0.896	92.1%	0.758	84.4%	0.783	84.0%
0.878	78.5%	0.882	74.60%	0.737	79.4%	0.810	85.7%	0.663	80.8%	0.744	78.8%
0.871	72.0%	0.871	87.30%	0.730	78.4%	0.792	86.8%	0.617	80.6%	0.697	80.0%
0.841	77.6%	0.866	77.80%	0.640	80.0%	0.726	77.8%	0.600	75.6%	0.667	70.4%
0.816	75.7%	0.866	74.60%	0.407	75.9%	0.675	76.7%	0.583	78.6%	0.667	65.7%

Model testing results for a model with equal sample sizes compared to the general model (a sex-agnostic model including all patients) as well as the female and male patients within the general model. AUROC and accuracy are provided for five different splits of the dataset

## Discussion

The maximum AAA diameter is the currently accepted criterion clinicians use for deciding whether to surgically intervene in AAAs to prevent rupture. However, this one-size-fits-all, one-dimensional metric does not always accurately predict which AAAs are likely to rupture, particularly for female patients. ML models have shown promise in predicting AAA outcomes with more discernability than the maximum diameter [24]. In this work, we used data from two different medical centers to explore different methods to address the disparity in outcome prediction between age-matched male and female AAA patients using ML classification models. This was achieved by creating sex-specific models for female and male patients and by equalizing the sample sizes between the groups. The output of these models was compared to that of a general ML model, constructed using patients of both sexes. Female patients had lower mean and peak wall stresses, smaller maximum diameters and maximum intraluminal thrombus thicknesses, and more symmetric lumens and walls.

It has been shown in literature that maximum transverse diameter, the currently accepted clinical means to predict AAA risk of rupture, has an accuracy of 73% and an AUROC of 0.741^16^. For this cohort, the accuracy of maximum transverse diameter alone was 68% and the AUROC was 0.537. Our ML models presented here all had accuracies and AUROCs greater than those values, suggesting that the ML models outperform maximum transverse diameter alone in predicting AAA patient outcome. Female patients and male patients experienced improved predictions using their respective sex-specific models compared to using the sex-agnostic general model. Feature importance for the male and general models included more biomechanical parameters in the top 15 features as compared to the female model. For this cohort, creating models with equal sample sizes between female and male patients led to improved predictions for both male and female patients.

Sex differences in AAA biomechanics and morphology have been previously demonstrated. However, many of the papers discussing sex differences in AAA acknowledge limitations in sample size and the need for further investigation with larger cohorts of patients. Female patients with AAAs have been shown to have lumens of smaller length and diameter than their male counterparts [33]. Additionally, female patients have been shown to have smaller ILT volume than male patients [33, 34]. While female patients have been shown to have lower elastin content than age- and diameter-matched males [35], a separate study that did not age- or diameter-match found no difference [36]. Progesterone and 17β-estradiol have been demonstrated to increase elastin deposition and decrease collagen deposition at a higher rate than testosterone in human aortic smooth muscle cells [37]. Our group showed a trend towards lower wall tissue tensile strength in female AAA compared to male AAA [38]. Female sex has also been shown to be a contributing factor in increased biomechanical risk of rupture in AAA [39].

Previous work has investigated using ML classification models to predict AAA outcomes. We recently demonstrated the utility of biomechanical and morphological indices in addition to clinical indices in predicting AAA outcome, but did not include any sex-based differences in those analyses [24]. Lindquist et al. 2021 found significant differences between male and female patients and included patient sex as a covariate in their model, but only had 35 female patients total in their analysis and did not explore sex-specific classification models [23]. Forneris et al. 2023 used a classifier model to predict accelerated AAA growth, but also had a small cohort size for their study [40].

There are multiple limitations in this proof-of-concept study. Due to the small sample size of repair and rupture cases for female patients, the repair and rupture groups were pooled for this study, unlike in our earlier sex-agnostic study [24]. Pooling repair and rupture cases assumes that these aneurysms would have ruptured since the clinician found the risk of rupture to be greater than the risks associated with surgery, but there is no way to know the eventual outcome of a repaired aneurysm if it did not undergo intervention. This may impact the generalizability of the models. To make sure the findings were consistent, the data were split multiple times to test multiple models and holdout testing was performed, but expanding this dataset may result in modified findings. A general model that predicted three outcomes as compared to two (Supplementary Table 1) was trained and tested, but due to smaller sample sizes in the sex-specific models, we were unable to consistently replicate AUROC values for some of the groups, such as the female rupture group. For the equal sample sizes model, findings may have been impacted by the smaller sample sizes and more similar diameters between the groups. Additionally, this dataset consists largely of patients of Caucasian descent, which is representative of the populations of the Pittsburgh, Pennsylvania and Eau Claire, Wisconsin metropolitan areas [40], but limits application to other geographical areas. Diversifying our dataset would allow for more generalizability of the model. Finally, we did not examine longitudinal data in this study. All CT scans analyzed were the first available for each patient, some of which were not clinically sized at this initial scan. As previous studies have suggested that AAA growth rate is correlated with rupture [4, 41] and is higher in women than men [42] and prediction of AAA growth has been demonstrated using deep learning [43, 44], adding a temporal aspect to this study could strengthen understanding of why certain AAA rupture compared to others.

Historically, female patients have been underrepresented in studies of AAA biomechanics. Although female patients experience lower prevalence rate of AAA than male patients [3], they experience AAA rupture rates at a rate exceeding that of their male counterparts [5]. ML shows promise in innovating how clinicians predict whether an aneurysm will be stable or need intervention [24], but it is important to consider how the model predicts based on sex. In this proof-of-concept study, we investigated whether creating sex-specific ML classification models improves prediction for female and male patients. It was found that a ML model trained on only female patients places less importance on biomechanical parameters than one trained on male patients or a general model. Additionally, for our dataset, training a model on only female patients or only male patients leads to improved predictions as compared to training a model on a dataset consisting of both male and female patients. In a clinical application, this tool could provide clinicians with a better picture as to the state of an AAA. These ML models outperform diameter in predicting AAA outcome [45]. However, as increased AAA prevalence for males leads to greater representation of male patients in datasets, it is important to consider the impact this has on predictions for female patients. Through sex-specific models or equalized sample sizes, bias in these models can be minimalized.

## Supplementary Information

Supplementary Material 1

## Data Availability

The datasets generated and/or analyzed during the current study are not publicly available due to limitations in scope of the IRB but are available from the corresponding author on reasonable request.

## References

[1] Brown LC, Powell JT. Risk factors for aneurysm rupture in patients kept under ultrasound surveillance. Ann Surg. 1999;230:289. 10493476 10.1097/00000658-199909000-00002 PMC1420874

[2] Robinson WP, Schanzer A, Li Y, Goodney PP, Nolan BW, Eslami MH, Cronenwett JL, Messina LM. Derivation and validation of a practical risk score for prediction of mortality after open repair of ruptured abdominal aortic aneurysms in a US regional cohort and comparison to existing scoring systems. J Vasc Surg. 2013;57:354–61. 23182157 10.1016/j.jvs.2012.08.120 PMC3773208

[3] Sciria CT, Osorio B, Wang J, Lu DY, Amin N, Vohra A, et al. Sex-based disparities in outcomes with abdominal aortic aneurysms. Am J Cardiol. 2021;155:135–48. 34294407 10.1016/j.amjcard.2021.06.023

[4] Brown PM, Zelt DT, Sobolev B. The risk of rupture in untreated aneurysms: the impact of size, gender, and expansion rate. J Vasc Surg. 2003;37:280–4. 12563196 10.1067/mva.2003.119

[5] Sweeting MJ, Thompson SG, Brown LC, Powell JT. Meta-analysis of individual patient data to examine factors affecting growth and rupture of small abdominal aortic aneurysms. Br J Surg. 2012;99:655–65. 22389113 10.1002/bjs.8707

[6] Powell JT, Brady AR, Brown LC, Fowkes FG, Greenhalgh RM, Ruckley CV, Thompson SG. Long-term outcomes of immediate repair compared with surveillance of small abdominal aortic aneurysms. N Engl J Med. 2002;346:1445–52. 12000814 10.1056/NEJMoa013527

[7] Mofidi R, Goldie VJ, Kelman J, Dawson ARW, Murie JA, Chalmers RT. A. Influence of sex on expansion rate of abdominal aortic aneurysms. Br J Surg. 2007;94:310–4. 17262754 10.1002/bjs.5573

[8] Li B, Eisenberg N, Witheford M, Lindsay TF, Forbes TL, Roche-Nagle G. Sex differences in outcomes following ruptured abdominal aortic aneurysm repair. JAMA Netw Open. 2022;5:e2211336–2211336. 35536576 10.1001/jamanetworkopen.2022.11336 PMC9092206

[9] Ulug P, Sweeting MJ, von Allmen RS, Thompson SG, Powell JT. Morphological suitability for endovascular repair, non-intervention rates, and operative mortality in women and men assessed for intact abdominal aortic aneurysm repair: systematic reviews with meta-analysis. Lancet. 2017;389:2482–91. 28455148 10.1016/S0140-6736(17)30639-6 PMC5483509

[10] Malayala SV, Raza A, Vanaparthy R. Gender-based differences in abdominal aortic aneurysm rupture: a retrospective study. J Clin Med Res. 2020;12:794–802. 33447313 10.14740/jocmr4376 PMC7781278

[11] Corsi T, Ciaramella MA, Palte NK, Carlson JP, Rahimi SA, Beckerman WE. Female sex is associated with reintervention and mortality following elective endovascular abdominal aortic aneurysm repair. J Vasc Surg. 2022;76:1494–e15011491. 35705120 10.1016/j.jvs.2022.05.011

[12] Chaikof EL, Dalman RL, Eskandari MK, Jackson BM, Lee WA, Mansour MA, Mastracci TM, Mell M, Murad MH, Nguyen LL. The Society for Vascular Surgery practice guidelines on the care of patients with an abdominal aortic aneurysm. *Journal of vascular surgery* 67, 2–77. e72 (2018). 10.1016/j.jvs.2017.10.044 29268916

[13] Darling R. Autopsy study of unoperated abdominal aortic aneurysms. The case for early resection. Circulation. 1977;56:161–4. 884821

[14] Vorp DA. Biomechanics of abdominal aortic aneurysm. J Biomech. 2007;40:1887–902. 17254589 10.1016/j.jbiomech.2006.09.003 PMC2692528

[15] Kontopodis N, Pantidis D, Dedes A, Daskalakis N, Ioannou CV. The - Not so - solid 5.5 cm threshold for abdominal aortic aneurysm repair: facts, misinterpretations, and future directions. Front Surg. 2016;3:1. 26835458 10.3389/fsurg.2016.00001 PMC4725249

[16] Singh TP, Moxon JV, Gasser TC, Jenkins J, Bourke M, Bourke B, Golledge J. Association between aortic peak wall stress and rupture index with abdominal aortic aneurysm-related events. Eur Radiol. 2023;33:5698–706. 36897345 10.1007/s00330-023-09488-1 PMC10326087

[17] Lo RC, Lu B, Fokkema MT, Conrad M, Patel VI, Fillinger M, Matyal R, Schermerhorn ML. Relative importance of aneurysm diameter and body size for predicting abdominal aortic aneurysm rupture in men and women. J Vasc Surg. 2014;59:1209–16. 24388278 10.1016/j.jvs.2013.10.104 PMC4004688

[18] Jones GT, Sandiford P, Hill GB, Williams MJA, Khashram M, Tilyard MW, et al. Correcting for body surface area identifies the true prevalence of abdominal aortic aneurysm in screened women. Eur J Vasc Endovasc Surg. 2019;57:221–8. 30293889 10.1016/j.ejvs.2018.08.048

[19] Nyrønning L, Skoog P, Videm V, Mattsson E. Is the aortic size index relevant as a predictor of abdominal aortic aneurysm? A population-based prospective study: the Tromsø study. Scand Cardiovasc J. 2020;54:130–7. 31909634 10.1080/14017431.2019.1707864

[20] Vande Geest JP, Di Martino ES, Bohra A, Makaroun MS, Vorp DA. A biomechanics-based rupture potential index for abdominal aortic aneurysm risk assessment: demonstrative application. Ann N Y Acad Sci. 2006;1085:11–21. 17182918 10.1196/annals.1383.046

[21] Gasser TC, Nchimi A, Swedenborg J, Roy J, Sakalihasan N, Böckler D. Hyhlik-Dürr, A. A novel strategy to translate the Biomechanical rupture risk of abdominal aortic aneurysms to their equivalent diameter risk: method and retrospective validation. Eur J Vasc Endovasc Surg. 2014;47:288–95. 24456739 10.1016/j.ejvs.2013.12.018

[22] Doyle BJ, Bappoo N, Syed MBJ, Forsythe RO, Powell JT, Conlisk N, Hoskins PR, McBride OMB, Shah ASV, Norman PE, Newby DE. Biomechanical assessment predicts aneurysm related events in patients with abdominal aortic aneurysm. Eur J Vasc Endovasc Surg. 2020;60:365–73. 32253165 10.1016/j.ejvs.2020.02.023

[23] Lindquist Liljeqvist M, Bogdanovic M, Siika A, Gasser TC, Hultgren R, Roy J. Geometric and biomechanical modeling aided by machine learning improves the prediction of growth and rupture of small abdominal aortic aneurysms. Sci Rep. 2021;11:18040. 34508118 10.1038/s41598-021-96512-3 PMC8433325

[24] Chung TK, Gueldner PH, Aloziem OU, Liang NL, Vorp DA. An artificial intelligence based abdominal aortic aneurysm prognosis classifier to predict patient outcomes. Sci Rep. 2024;14:3390. 38336915 10.1038/s41598-024-53459-5 PMC10858046

[25] Chung TK, Liang NL, Vorp DA. Artificial intelligence framework to predict wall stress in abdominal aortic aneurysm. Appl Eng Sci. 2022. 10.1016/j.apples.2022.100104 37711641 10.1016/j.apples.2022.100104 PMC10500563

[26] Chung TK, Gueldner PH, Kickliter TM, Liang NL, Vorp DA. An objective and repeatable sac isolation technique for comparing biomechanical metrics in abdominal aortic aneurysms. Bioengineering. 2022;9:601. 36354512 10.3390/bioengineering9110601 PMC9687639

[27] Zhu L, Kukko A, Virtanen J-P, Hyyppä J, Kaartinen H, Hyyppä H, et al. Multisource point clouds, point simplification and surface reconstruction. Remote Sens. 2019;11:2659.

[28] Di Martino ES, Vorp DA. Effect of variation in intraluminal thrombus constitutive properties on abdominal aortic aneurysm wall stress. Ann Biomed Eng. 2003;31:804–9. 12971613 10.1114/1.1581880

[29] Wang DH, Makaroun M, Webster MW, Vorp DA. Mechanical properties and microstructure of intraluminal thrombus from abdominal aortic aneurysm. J Biomech Eng. 2001;123:536–9. 11783723 10.1115/1.1411971

[30] Vande Geest JP, Schmidt DE, Sacks MS, Vorp DA. The effects of anisotropy on the stress analyses of patient-specific abdominal aortic aneurysms. Ann Biomed Eng. 2008;36:921–32. 18398680 10.1007/s10439-008-9490-3 PMC2674610

[31] Lundberg SM, Lee S-I. A unified approach to interpreting model predictions. Adv Neural Inf Process Syst. 2017;30:1–10.

[32] Jeon BB, Kottakota AK, Kerr KE, Gueldner PH, Guffey MJ, Sen I, Liang NL, Vorp DA, Chung. Optimizing Predictive Performance Without Sacrificing Explainability: Comparing Logistic Regression and Ensemble Decision Trees for Abdominal Aortic Aneurysm Repair Outcomes, JVS-Vasc Insights. 2025. 10.1016/j.jvsvi.2025.100300. 10.1016/j.jvsvi.2025.100300 PMC13568067 42730357

[33] Gao Z, Xiong J, Chen Z, Deng X, Xu Z, Sun A, Fan Y. Gender differences of morphological and hemodynamic characteristics of abdominal aortic aneurysm. Biol Sex Differ. 2020;11:41. 32693818 10.1186/s13293-020-00318-3 PMC7372899

[34] Yasuhara H, Ohara N, Nagawa H. Influence of gender on intraluminal thrombus of abdominal aortic aneurysms. Am J Surg. 2001;182:89–92. 11532424 10.1016/s0002-9610(01)00653-5

[35] Villard C, Eriksson P, Swedenborg J, Hultgren R. Differences in elastin and elastolytic enzymes between men and women with abdominal aortic aneurysm. Aorta (Stamford). 2014;2:179–85. 26798738 10.12945/j.aorta.2014.14-017 PMC4686357

[36] Tong J, Schriefl AJ, Cohnert T, Holzapfel GA. Gender differences in biomechanical properties, thrombus age, mass fraction and clinical factors of abdominal aortic aneurysms. Eur J Vasc Endovasc Surg. 2013;45:364–72. 23395130 10.1016/j.ejvs.2013.01.003

[37] Natoli AK, Medley TL, Ahimastos AA, Drew BG, Thearle DJ, Dilley RJ, Kingwell BA. Sex steroids modulate human aortic smooth muscle cell matrix protein deposition and matrix metalloproteinase expression. Hypertension. 2005;46:1129–34. 16230520 10.1161/01.HYP.0000187016.06549.96

[38] VANDE GEEST JP, DILLAVOU ED, MARTINO DI, OBERDIER ES, MAKAROUN MBOHRAA, M. S., VORP DA. Gender-Related differences in the tensile strength of abdominal aortic aneurysm. Ann N Y Acad Sci. 2006;1085:400–2. 17182963 10.1196/annals.1383.048

[39] Lindquist Liljeqvist M, Hultgren R, Siika A, Gasser TC, Roy J. Gender, smoking, body size, and aneurysm geometry influence the Biomechanical rupture risk of abdominal aortic aneurysms as estimated by finite element analysis. J Vasc Surg. 2017;65:1014–e10211014. 28342508 10.1016/j.jvs.2016.10.074

[40] Forneris A, Beddoes R, Benovoy M, Faris P, Moore RD, Di Martino E. AI-powered assessment of biomarkers for growth prediction of abdominal aortic aneurysms. JVS-Vascular Science. 2023;4:100119. 37662586 10.1016/j.jvssci.2023.100119 PMC10470267

[41] Lederle FA, Johnson GR, Wilson SE, Ballard DJ, Jordan WD Jr., Blebea J, Littooy FN, Freischlag JA, Bandyk D, Rapp JH, Salam AA. Rupture rate of large abdominal aortic aneurysms in patients refusing or unfit for elective repair. JAMA. 2002;287:2968–72. 12052126 10.1001/jama.287.22.2968

[42] Hannawa KK, Eliason JL, Upchurch GR Jr. Gender differences in abdominal aortic aneurysms. Vascular. 2009;17(Suppl 1):S30-39. 10.2310/6670.2008.00092 PMC2913052 19426607

[43] Kim S, Jiang Z, Zambrano BA, Jang Y, Baek S, Yoo S, Chang HJ. Deep learning on multiphysical features and hemodynamic modeling for abdominal aortic aneurysm growth prediction. IEEE Trans Med Imaging. 2023;42:196–208. 36094984 10.1109/TMI.2022.3206142

[44] Akkoyun E, Kwon ST, Acar AC, Lee W, Baek S. Predicting abdominal aortic aneurysm growth using patient-oriented growth models with two-step bayesian inference. Comput Biol Med. 2020;117:103620. 32072970 10.1016/j.compbiomed.2020.103620 PMC7064358

[45] Fillinger MF, Marra SP, Raghavan ML, Kennedy FE. Prediction of rupture risk in abdominal aortic aneurysm during observation: wall stress versus diameter. J Vasc Surg. 2003;37:724–32. 12663969 10.1067/mva.2003.213

