# Supplementary material for: Sex-specific machine learning classification models improve outcome prediction for abdominal aortic aneurysms
Source: Biol Sex Differ. 2025 Nov 11;16:96. doi: 10.1186/s13293-025-00765-w (PMC12607067; doi:10.1186/s13293-025-00765-w)
Supplement: Supplementary file 1 — Supplementary Material 1 [file 13293_2025_765_MOESM1_ESM.pptx]

## Slide 1
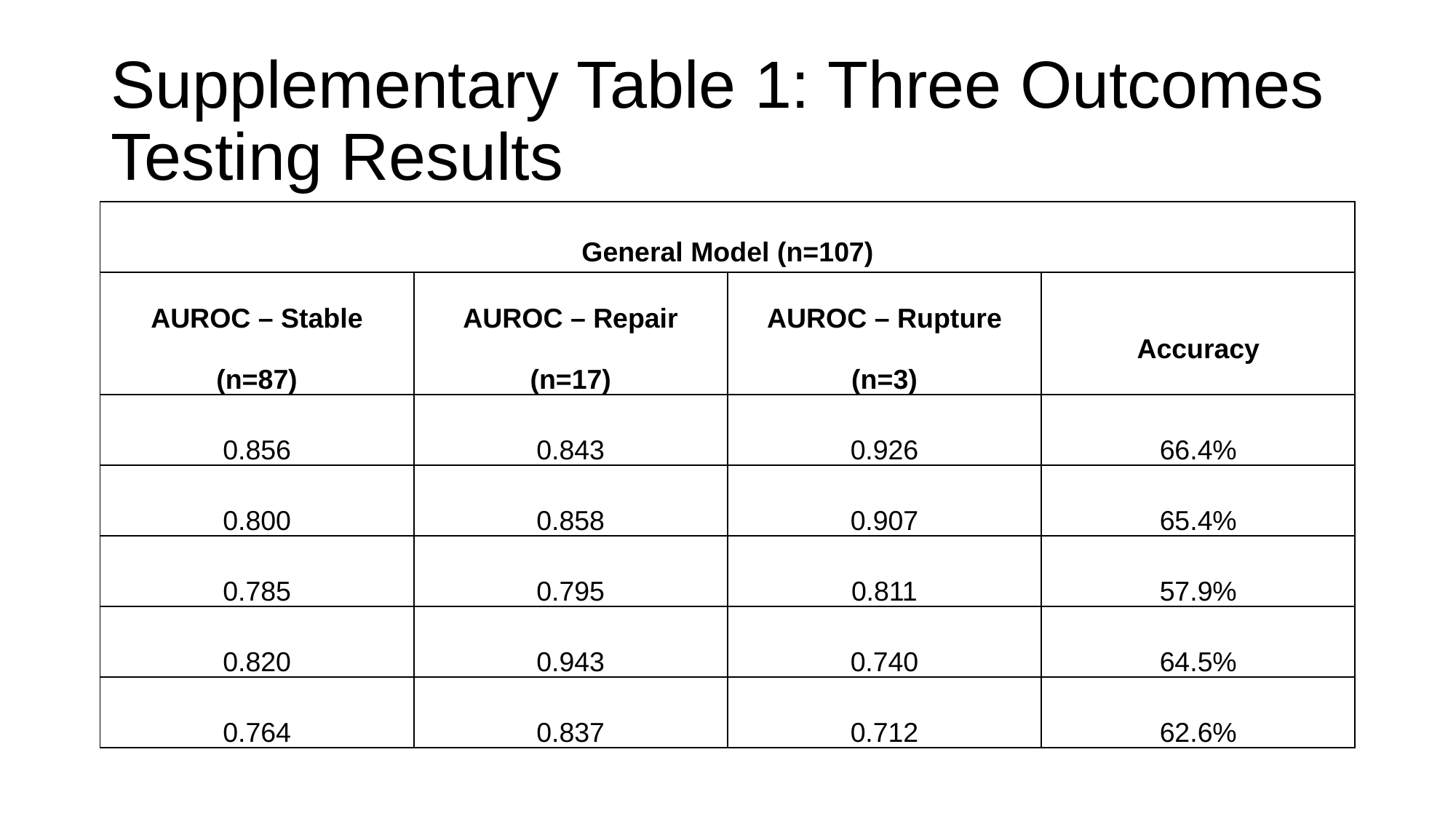

# Supplementary Table 1: Three Outcomes Testing Results
| General Model (n=107) | | | |
| --- | --- | --- | --- |
| AUROC – Stable (n=87) | AUROC – Repair (n=17) | AUROC – Rupture (n=3) | Accuracy |
| 0.856 | 0.843 | 0.926 | 66.4% |
| 0.800 | 0.858 | 0.907 | 65.4% |
| 0.785 | 0.795 | 0.811 | 57.9% |
| 0.820 | 0.943 | 0.740 | 64.5% |
| 0.764 | 0.837 | 0.712 | 62.6% |

## Slide 2
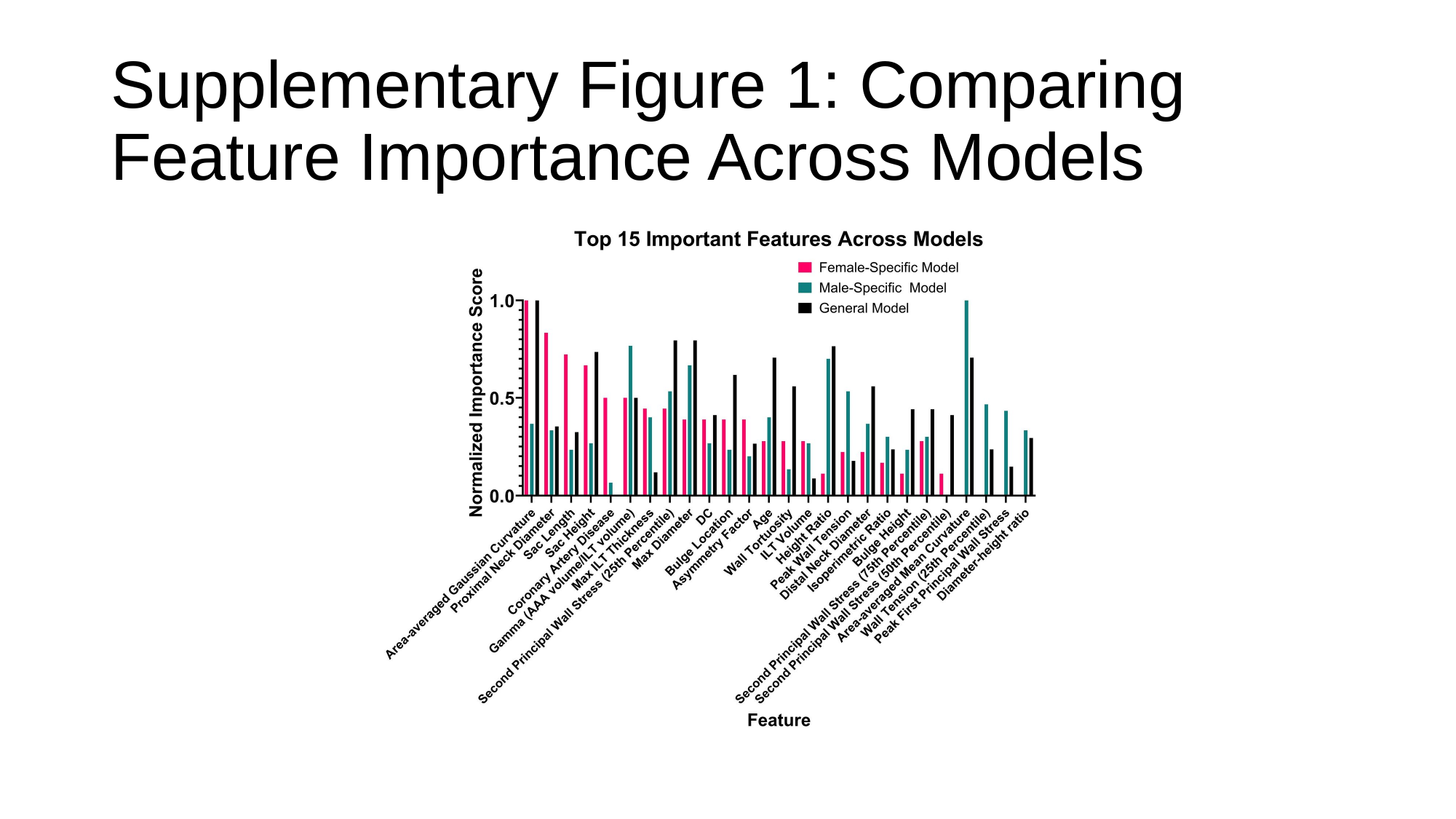

# Supplementary Figure 1: Comparing Feature Importance Across Models
